# Characterization of AtBAG2 as a Novel Molecular Chaperone

**DOI:** 10.3390/life13030687

**Published:** 2023-03-03

**Authors:** Chang Ho Kang, Jae Hyeok Lee, Yeon-Ju Kim, Cha Young Kim, Soo In Lee, Jong Chan Hong, Chae Oh Lim

**Affiliations:** 1 Division of Applied Life Sciences (BK21+), Plant Molecular Biology and Biotechnology Research Center, Gyeongsang National University, Jinju 52828, Republic of Korea; 2 Graduate School of Biotechnology, College of Life Science, Kyung Hee University, Yongin-si 17104, Republic of Korea; 3 Korean Collection for Type Cultures, Biological Resource Center, Korea Research Institute of Bioscience and Biotechnology (KRIBB), Jeongeup 56212, Republic of Korea; 4 Department of Agricultural Biotechnology, National Institute of Agricultural Sciences (NAS), Rural Development Administration (RDA), Jeonju 54874, Republic of Korea

**Keywords:** BAG (Bcl-2-associated anthanogene) family proteins, abiotic stress, molecular chaperone

## Abstract

Bcl-2-associated anthanogene (BAG) family proteins regulate plant defense against biotic and abiotic stresses; however, the function and precise mechanism of action of each individual BAG protein are not yet clear. In this study, we investigated the biochemical and molecular functions of the *Arabidopsis thaliana* BAG2 (AtBAG2) protein, and elucidated its physiological role under stress conditions using mutant plants and transgenic yeast strains. The T-DNA insertion *atbag2* mutant plants were highly susceptible to heat shock, whereas transgenic yeast strains ectopically expressing *AtBAG2* exhibited outstanding thermotolerance. Moreover, a biochemical analysis of GST-fused recombinant proteins produced in bacteria revealed that AtBAG2 exhibits molecular chaperone activity, which could be attributed to its BAG domain. The relevance of the molecular chaperone function of AtBAG2 to the cellular heat stress response was confirmed using yeast transformants, and the experimental results showed that overexpression of the *AtBAG2* sequence encoding only the BAG domain was sufficient to impart thermotolerance. Overall, these results suggest that the BAG domain-dependent molecular chaperone activity of AtBAG2 is indispensable for the heat stress response of Arabidopsis. This is the first report demonstrating the role of AtBAG2 as a sole molecular chaperone in Arabidopsis.

## 1. Introduction

B cell lymphoma 2 (Bcl2)-associated athanogene (BAG) family proteins are conserved across a wide range of eukaryotes including animals, yeast, and plants. BAG1, the first BAG protein to be discovered, was identified by screening for human Bcl-2-interacting proteins in a mouse embryo cDNA library [1,2,3]. To date, six BAG proteins (BAG1–BAG6), harboring a conserved BAG domain (BD) at the C-terminus have been reported in humans [4]. The three-dimensional structure of the BD, which contains approximately 110 amino acids (aa), comprises three α-helices, each containing 30–40 aa [5,6]. Through the BD, BAG proteins modulate the refolding activity of the heat shock-inducible heat shock protein 70 (HSP70) and the constitutively expressing heat shock cognate protein 70 (HSC70) by interacting with their ATPase domain [5]. In mammals, BAG proteins interact with members of diverse protein families to regulate various cellular phenomena such as protein degradation and cell apoptosis, migration, and proliferation [7,8].

In plants, *BAG* genes are associated with development and stress responses [9]. An advanced bioinformatics analysis revealed that the model plant *Arabidopsis thaliana* contains seven BAG proteins (AtBAG1–AtBAG7) [10,11]. Among these, AtBAG1–AtBAG4 carry an N-terminal ubiquitin-like (UBL) domain, whereas AtBAG5–AtBAG7 are plant-specific proteins harboring a calmodulin (CaM)-binding motif near the BD [10,11]. AtBAG proteins are reportedly involved in various processes related to plant development and environmental stress response. For example, AtBAG1 plays a critical role in the Hsc70-mediated proteasomal degradation of misfolded and unimported plastid proteins in the cytosol, and an optimal level of this protein is important for normal plant growth. AtBAG6, a CaM-binding protein, induces programmed cell death (PCD) in Arabidopsis and yeast, and its 134 aa stretch, which encompasses both the CaM-binding IQ motif and BD, is sufficient to induce cell death [11]. Additionally, AtBAG4, AtBAG6, and AtBAG7 are involved in the plant response to pathogen attack as well as the tolerance to cold, heat, ultraviolet (UV), and endoplasmic reticulum (ER) stresses [9,10,12]. Recently, *AtBAG2* and *AtBAG6* were reported to be involved in the tolerance to multiple abiotic stresses in Arabidopsis [13]. However, information on the biochemical and molecular functions of BAG proteins that affect plant physiology is extremely limited. In this study, we explored the intrinsic biochemical and molecular properties of AtBAG2 and characterized this protein as a novel molecular chaperone.

## 2. Materials and Methods

### 2.1. Plant Material and Growth Conditions

*Arabidopsis thaliana* ecotype Columbia (Col-0) was used as the wild type (WT) in this study. The homozygous T-DNA mutant *atbag2* (SALK_030295; Col-0 background) was obtained from the Arabidopsis Biological Resource Center (ABRC; Ohio State University, Columbus, OH, USA). Seeds were sown in Petri dishes containing full-strength Murashige and Skoog (MS) medium (Duchefa Biochemie B.V., Haarlem, The Netherlands) supplemented with 2% (*w*/*v*) sucrose and 1% (*w*/*v*) agar, and cold-stratified in the dark at 4 °C for 3 days. The plates were then transferred to an environmentally controlled growth chamber maintained at 22 °C temperature, a 16 h light/8 h dark photoperiod, and 100 μmol m^−2^ s^−1^ light intensity. The chemicals were purchased from Sigma-Aldrich (Sigma-Aldrich Chemical, Saint Louis, MO, USA).

### 2.2. Heat Shock Treatment and Phenotypic Analysis of Plants

To examine the thermotolerance of the WT and *atbag2* mutant, seedlings were grown on solid nutrient medium containing 2% (*w*/*v*) sucrose, and incubated under the abovementioned conditions [14]. Plates containing 7-day-old seedlings were sealed with plastic electrical tape and submerged for 30 min in a water bath maintained at 44 °C. The plates were then transferred to normal growth conditions, and the thermotolerance of seedlings was determined by measuring their survival rate. To measure electrolyte leakage, the seedlings were submerged in 5 mL of deionized water and placed on a shaker at 22 °C. The initial conductivity of the solution was measured using an Orion 3-Star Benchtop Conductivity Meter (Thermo Electron Cooperation, Rosemount, MN, USA). The seedlings were then autoclaved for 15 min, and conductivity was measured again (final conductivity) to determine the total amount of ions in solution. Percent ion leakage was estimated as the ratio of initial conductivity to final conductivity.

### 2.3. Yeast Strain, Survivability Assay, and TB Exclusion Assay

The yeast (*Saccharomyces cerevisiae*) strain W303 (SVL82; *MaTα*, *ade2*, *his3*, *leu2*, *trp1*, *ura3*, *can1*) was grown in Yeast Extract-Peptone-Dextrose (YPD) medium at 27 °C [15]. Cells transformed with the glutathione S-transferase (GST)-only vector (control) and various GST-AtBAG2 fusions (G-AtBAG2, G-AtBAG2-Nt, G-AtBAG2-M, and G-AtBAG2-Ct) were grown in the YPD medium overnight. The cells were then transferred to fresh YPD medium and incubated at a concentration of 5 × 107 cells/mL at 27 °C or 55 °C. Cells were sampled at 0, 10, 20, 30, 40, 50, and 60 min after incubation, and suitable dilutions were plated on YPD agar plates. The number of viable cells or colony forming units (CFU) was counted after 2–3 days of incubation at 27 °C.

Samples of vector, *G-AtBAG2*, *G-AtBAG2-Nt*, *G-AtBAG2-M*, and *G-AtBAG2-Ct* transformants collected at the 60 min time point were subjected to trypan blue (TB) staining and spot assays. The TB staining assay was performed as described previously [16]. To conduct spot assays, the concentration of each culture was adjusted to an optical density (OD_600_) of 1.0. Next, 6 μL aliquots of 10-fold serial dilutions were spotted onto YPD agar plates, incubated at 27 °C, and examined after 2–3 days.

### 2.4. Construction of Expression Plasmids

The *AtBAG2* coding sequence was PCR-amplified using the following primer pair: AtBAG2(*Bam*HI)-F (5′-GGATCCTAATGATGAAAATGAGTATCGGA-3′) and AtBAG2(*Xho*I)-R (5′-CTCGAGTTAATTGAATAATTCCCATTTA-3′). The PCR products were cloned into the pGEM-T Easy vector (Promega, Madison, WI, USA), and the *Bam*HI/*Xho*I fragments were subcloned into the pGEX-2T vector (GE Healthcare, Piscataway, NJ, USA). To identify the amino acid sequence required for the molecular chaperone activity of AtBAG2 and the thermotolerance of yeast cells, deletion fragments of *AtBAG2* were generated by PCR using the following primers: AtBAG2(*Bam*HI)-F and AtBAG2-Nt(*Xho*I)-R (5′-CTCGAGTTAGATTGCTTTAGATGATTT-3′) for *AtBAG2-Nt* (1–133 aa); AtBAG2-M(*Bam*HI)-F (5′-GGATCCTAATGAAAGAGAAATCATCTAAA-3′) and AtBAG2-M(*Xho*I)-R (5′-CTCGAGTTACTGCATCTTCTTCTTCAA-3′) for *AtBAG2-M* (131–195 aa); and AtBAG2-Ct(*Bam*HI)-F (5′-GGATCCTAATGAAGAAGAAGATGCAGAAT-3′) and At-BAG2(*Xho*I)-R for *AtBAG2-Ct* (191–296 aa). The amplified products were cloned into the pGEM-T Easy vector, and subcloned into the pGEX-2T expression vector digested with *Bam*HI and *Xho*I (for expression in *E. coli*) or into the pYES2-GST fusion vector digested with *Bam*HI and *Xho*I (for expression in yeast).

### 2.5. Recombinant Protein Production and Purification

The pGEX-AtBAG2, pGEX-AtBAG2-Nt, pGEX-AtBAG2-M, and pGEX-AtBAG2-Ct vectors were transformed into *Escherichia coli* BL21(DE3) pLysS cells. Recombinant protein production and purification were performed according to the procedure described previously [17]. The AtBAG2 deletion variants were further purified using the TSK heparin-5PW HPLC column (7.5 mm × 75 mm), as described previously [17,18]. DnaK, a possible co-purifying contaminant on GSH columns, was removed using the ATP-agarose column (Amersham Pharmacia Biotech, Piscataway, NJ, USA), according to the manufacturer’s instructions. The purified AtBAG2 proteins were dialyzed against 20 mM HEPES (pH 8.0) before use.

### 2.6. Protein Quantification and Chaperone Activity Assay

Mixtures containing a given recombinant AtBAG2 protein (30 μg/mL protein in 20 mM HEPES [pH 8.0]) and 10 μM 4,4′-bis (1-anilinonaphthalene 8-sulfonate) (bis-ANS; Sigma-Aldrich) were incubated at various temperatures for 30 min. The fluorescence of each mixture was then measured using a SFM25 spectrofluorometer (Kontron, Zurich, Switzerland) at an excitation wavelength of 380 nm and emission wavelengths ranging from 400–600 nm.

The chaperone activity of recombinant proteins was measured using citrate synthase (CS), malate dehydrogenase (MDH), and insulin (Sigma-Aldrich) as substrates, as described previously [19,20]. Turbidity caused by substrate aggregation was monitored using the DU800 spectrophotometer (Beckman Coulter, Indianapolis, IN, USA) equipped with a thermostatic cell holder.

## 3. Results

### 3.1. AtBAG2 Enhances Heat Shock Tolerance in Arabidopsis

**Figure 1:**
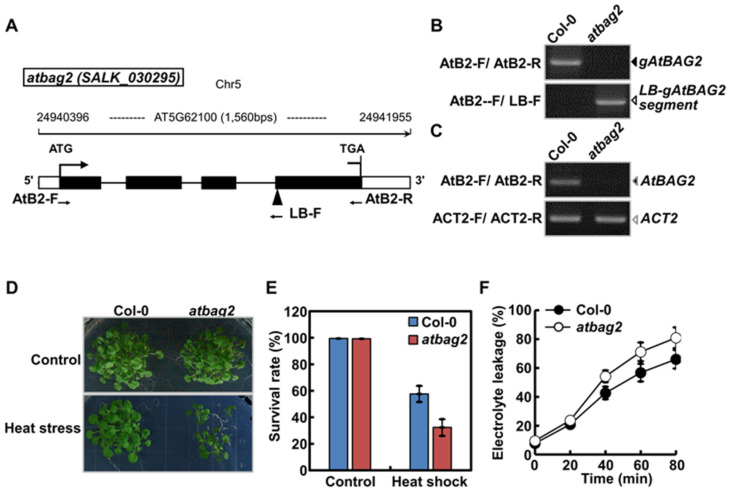
*AtBAG2* regulates heat stress tolerance in Arabidopsis. (**A**) Schematic representation of the predicted gene structure of *AtBAG2*. Black elbow arrows represent the start codon (ATG). Filled boxes and horizontal black lines indicate exons and introns, respectively. Empty boxes at either end of the gene represent 5′ and 3′ untranslated regions (UTRs). A black arrowhead indicates the T-DNA position in *atbag2*. Black arrows represent the binding sites of primers used for genotyping. (**B**,**C**) Confirmation of homozygous *atbag2* mutant lines by genomic DNA-based PCR (**B**) and sqRT-PCR (**C**). (**D**) Photographs of WT (Col-0) and *atbag2* seedlings grown at optimal temperature (22 °C) for 14 days (control) or subjected to heat stress conditions (7-day-old seedlings treated with heat shock [44 °C] for 30 min, and then grown at 22 °C for 7 days). (**E**,**F**) Comparison of survival rate (**E**) and ion leakage (**F**) between 7-day-old WT (Col-0) and *atbag2* seedlings subjected to the heat shock treatment, as described in (**D**). In (**E**), the survival rates of heat-stressed Col-0 and *atbag2* seedlings (heat shock) were compared with those of unstressed plants (control). Data represent the mean ± standard deviation (SD) of at least three independent experiments. To measure the ion leakage of Col-0 (-●-) and *atbag2* (-○-) seedlings shown in (**F**), samples were collected at the indicated times and submerged in deionized water for 1 day. The conductivity of at least 10 seedlings was measured before autoclaving (initial conductivity) and after autoclaving (final conductivity). Data represent the mean ± SD of at least three independent experiments.

To confirm the physiological significance of *AtBAG2* in plant stress response, we selected homozygous lines of the *atbag2* T-DNA insertion mutant originally obtained from ABRC (OH, USA) (Figure 1A,B, Figures S1 and S2). Transcript levels of *AtBAG2* were compared between WT (Col-0) and *atbag2* mutant plants by semi-quantitative reverse transcription PCR (sqRT-PCR) (Figure 1C, Figures S3 and S4). Although the *AtBAG2* signal was clearly detected in WT plants, it was barely detectable in the *atbag2* mutant, as expected (Figure 1C and Figure S3). By contrast, the signal for the *Actin-2* (*ACT2*) gene (*AT3G18780*), which was used as a control, was nearly identical in both WT and *atbag2* samples (Figure 1C and Figure S4). Next, we investigated the physiological function of *AtBAG2* in 7-day-old WT and *atbag2* seedlings under heat shock conditions (Figure 1D–F). Under normal conditions (22 °C), the two genotypes exhibited no difference in seed germination and seedling survival rates (Figure 1D,E). However, when incubated at 44 °C for 30 min, the 7-day-old *atbag2* seedlings exhibited outstanding thermosensitivity (Figure 1D,E), as indicated by their low survival rate (31.4 ± 5.6%) compared with the WT (56.6 ± 5.2%) (Figure 1E). Because heat shock causes considerable damage to the channel and transporter proteins, fluidity, and other properties of the cell membrane, resulting in electrolyte leakage [21,22], we compared ion leakage between the WT and *atbag2* plants. Following incubation at 44 °C for 30 min, the seedlings of both genotypes exhibited an increase in ion leakage; however, the level of ion leakage was higher in *atbag2* seedlings than in the WT (Figure 1F). The WT and *atbag2* seedlings took 53 and 37 min, respectively, to reach 50% ion leakage. Overall, these results indicate that *AtBAG2* plays a major role in plant survival under heat stress.

### 3.2. AtBAG2 Overexpression Enhances Thermotolerance in Yeast

**Figure 2:**
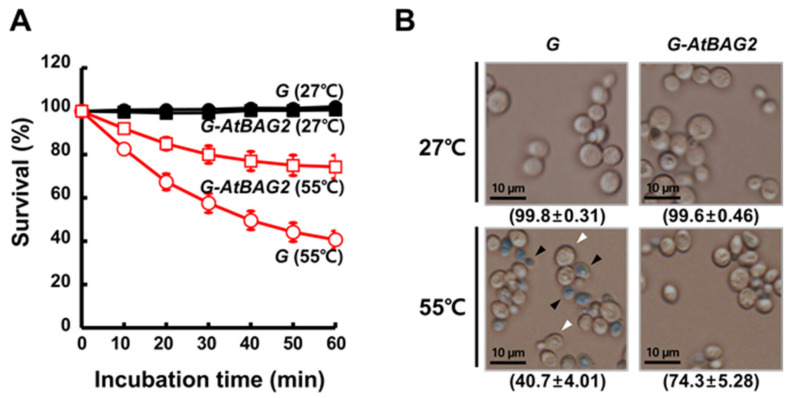
Ectopic expression of *AtBAG2* enhances the thermotolerance of yeast cells. (**A**) Effect of heat stress on yeast cell viability. Yeast cells transformed with pYES2-G-AtBAG2 (*G-AtBAG2*) or pYES2-G (*G*) were grown in YPD media supplemented with 2% galactose (Gal). The transformed cells (5 × 10^7^ cells/mL) were incubated at 27 °C or 55 °C and sampled at the indicated time points to count the number of viable cells. The cell survival rate (%) of each transformant was calculated as the ratio of the viable cell count at a given time point to the viable cell count at the 0 min time point. Data represent the mean ± SD of at least three independent experiments. (**B**) TB exclusion assay. Samples of *G-AtBAG2* and *G* transformants incubated at 55 °C for 1 h in (**A**) were visualized by fluorescence microscopy after staining with TB. White and black arrowheads indicate TB-negative and -positive cells, respectively. Scale bars, 10 μm. Data represent the mean ± SD of at least three independent experiments.

Because *atbag2* plants were more sensitive to heat shock than WT plants, we investigated whether *AtBAG2* imparts heat stress tolerance at the cellular level by monitoring the effects of *AtBAG2* overexpression in yeast cells. To conduct this experiment, we cloned the *GST*-*AtBAG2* gene fusion under the control of the *GAL1* promoter to generate the *pYES2-G-AtBAG2* construct. We then transformed *pYES2-G-AtBAG2* and the *GST (G)*-only construct (*pYES2-G*) separately into yeast W303 cells for the conditional overexpression of recombinant proteins. To confirm the physiological significance of *AtBAG2* overexpression in yeast, we compared the viability of heat-shocked *G* and *G-AtBAG2* transformants. Cultures of the two transformants were adjusted to equal cell densities at the mid-exponential growth stage, and aliquots were incubated at 27 °C or 55 °C. Viable cell counts were measured at regular intervals (Figure 2A). The *G*- and *G-AtBAG2*-transformed cells showed no significant difference in percent survival upon incubation at 27 °C for up to 60 min. However, at 55 °C, the survival rate of *G* transformants was dramatically lower than that of *G-AtBAG2* transformants (57.6 ± 4.73% and 80.9 ± 3.97%, respectively, at 30 min; 43.3 ± 4.22% and 73.6 ± 5.37%, respectively, at 60 min). To confirm these results, we performed the TB exclusion assay [23]; in this assay, only dead cells are expected to stain blue as they cannot exclude the dye. No TB-positive *G*- and *G-AtBAG2*-transformed cells were observed at 27 °C after 60 min (Figure 2B). However, after a 60 min incubation at 55 °C, more than 50% of the *G*-transformed cells appeared to be dead (intense blue staining), while the *G-AtBAG2* transformants were alive (mild blue staining) (Figure 2B). Taken together, our results consistently demonstrated that *G-AtBAG2* overexpression resulted in a remarkable increase in the thermotolerance of yeast cells.

### 3.3. AtBAG2 Functions as a Molecular Chaperone

**Figure 3:**
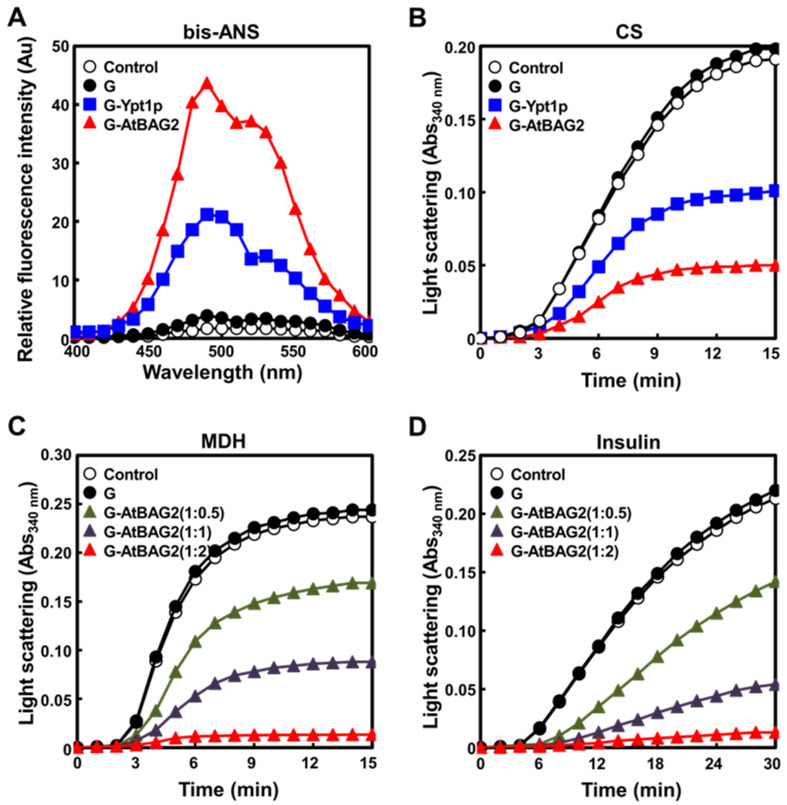
AtBAG2 acts as a molecular chaperone. (**A**) Results of bis-ANS-binding assay used to investigate the effect of heat shock on the hydrophobic domains of GST alone (G), GST-AtBAG2 fusion (G-AtBAG2), and G-Ypt1p fusion (G-Ypt1p; control). The fluorescence spectra of 10 μM bis-ANS (-○-) and 10 μM bis-ANS plus 30 μM G-AtBAG2 (-▲-), G-Ypt1p (-■-), or G (-●-) were measured at 380 nm (excitation wavelength) and 400–600 nm (emission wavelengths). (**B**–**D**) Molecular chaperone activity assay of AtBAG2 using CS (**B**), MDH (**C**), and insulin (**D**). Light scattering was monitored at 340 nm. In (**B**), 1 μM CS was incubated either alone (-○-) or with 2 μM G (-●-), G-Ypt1p (-■-), or G-AtBAG2 (-▲-) in 50 mM HEPES buffer (pH 7.0) in a spectrophotometer cell at 43 °C. In (**C**), 1.67 μM MDH was incubated alone (-○-), with 0.84 μM (-▲-), 1.67 μM (-▲-), or 3.34 μM (-▲-) G-AtBAG2, or with 3.34 μM G (-●-) in 50 mM HEPES buffer (pH 7.0) in a spectrophotometer cell at 45 °C. In (D), 1 μM insulin was incubated alone (-○-), with 0.5 μM (-▲-), 1 μM (-▲-), or 2 μM (-▲-) G-AtBAG2, or with 2 μM G (-●-) in 50 mM HEPES buffer (pH 8.0) containing 10 mM DTT in a spectrophotometer cell at 25 °C. Data represent the mean of at least three independent experiments.

Since *AtBAG2* conferred thermotolerance in both Arabidopsis and yeast, we explored the mechanism of action of the AtBAG2 protein by examining whether it exhibits molecular chaperone activity. To study the molecular chaperone activity of AtBAG2, we examined its surface hydrophobicity using bis-ANS, which binds to hydrophobic patches on proteins and fluoresces at an emission maximum of ~470 nm [24], and explored its ability to prevent the heat-induced aggregation of CS and MDH or dithiothreitol (DTT)-induced aggregation of insulin by measuring light scattering at 340 nm. The hydrophobicity of GST-fused AtBAG2 (G-AtBAG2) and its ability to prevent protein aggregation were compared with those of GST-fused Ypt1p (G-Ypt1p), a previously characterized molecular chaperone [19] (positive control), and GST alone (G; negative control). The fluorescence signal of bis-ANS-bound G-AtBAG2 was greater than that of G-Ypt1p (Figure 3A), suggesting that the chaperone activity of G-AtBAG2 is higher than that of G-Ypt1p. High surface hydrophobicity is a typical characteristic of molecular chaperones [25]. Based on these results, we investigated the chaperone activity of G-AtBAG2, along with those of G-Ypt1p (positive control) and G (negative control). In light scattering experiments, when CS (2 μM), a substrate protein [19,20], was heated alone or in the presence of 5-fold molar excess (8.35 μM) of the G protein, light scattering increased rapidly; however, when 2 μM CS was heated in the presence of 5-fold molar excess of G-AtBAG2 and G-Ypt1p, its aggregation was successively inhibited, confirming that G-AtBAG2 and G-Ypt1p function as molecular chaperones (Figure 3B). Interestingly, the aggregation prevention activity of G-AtBAG2 was greater than that of G-Ypt1p (Figure 3B). Furthermore, when MDH (1.67 μM) was heated alone or along with a two-fold molar excess (3.34 μM) of G, light scattering increased rapidly; however, when 1.67 μM MDH was heated in the presence of increasing amounts of G-AtBAG2 (0.5-, 1-, and 2-fold molar excess), its aggregation was inhibited in a concentration-dependent manner, confirming that G-AtBAG2 exhibits chaperone activity (Figure 3C). When G-AtBAG2 was present at two-fold molar excess (3.34 μM), approximately 90% of the MDH protein was protected against heat-induced aggregation during the 15 min incubation period (Figure 3C). G-AtBAG2 also inhibited the DTT-induced aggregation of insulin in a concentration-dependent manner (Figure 3D). These observations clearly demonstrate that AtBAG2 functions as a molecular chaperone.

### 3.4. BD Is Required for the Molecular Chaperone Activity of AtBAG2

**Figure 4:**
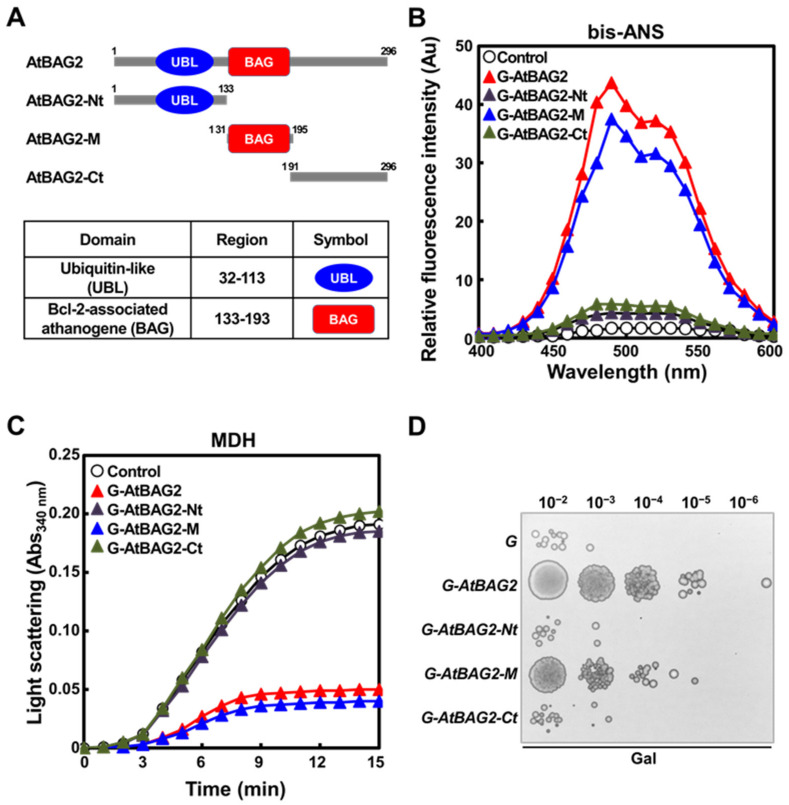
Identification of the functional domain responsible for the molecular chaperone activity of AtBAG2. (**A**) Schematic representation of AtBAG2 and its serial deletion variants (AtBAG2-Nt, -M, and -Ct). The circle and box indicate the UBL domain and BD, respectively. The amino acid positions of each deletion variant are indicated. G-AtBAG2 and G-AtBAG2-Nt, -M, and -Ct represent the four GST (G)-fusion constructs containing the indicated fragments of AtBAG2. (**B**) Results of bis-ANS-binding assays used to study the effect of heat shock on the hydrophobic domains of G-AtBAG2 and G-AtBAG2-Nt, -M, and -Ct. The samples used were 10 μM bis-ANS (-○-), 10 μM bis-ANS plus 30 μM G-AtBAG2 (-▲-), G-AtBAG2-Nt (-▲-), G-AtBAG2-M (-▲-), and G-AtBAG2-Ct (-▲-). (**C**) Molecular chaperone activity assay. Solutions containing either 1.67 μM MDH alone (-○-) or with 2.5 μM G-AtBAG2 (-▲-), G-AtBAG2-Nt (-▲-), G-AtBAG2-M (-▲-), and G-AtBAG2-Ct (-▲-) in 50 mM HEPES buffer (pH 7.0) were incubated in a spectrophotometer cell at 45 °C. (**D**) Yeast spot assay. Yeast cells transformed with pYES2-G-AtBAG2 (*G-AtBAG2*), pYES2-G-AtBAG2-Nt (*G-AtBAG2-Nt*), pYES2-G-AtBAG2-M (*G-AtBAG2-M*), pYES2-G-AtBAG2-Ct (*G-AtBAG2-Ct*), or pYES2-G (*G*) were grown in YPD media supplemented with 2% Gal. The transformed cells (5 × 10^7^ cells/mL) were incubated at 55 °C for 1 h. Next, 6 μL aliquots of 10-fold serial dilutions of each cell suspension were spotted on YPD plates. The plates were incubated at 27 °C for 3 days and then photographed.

The analysis of the protein domain structure on the InterPro website (http://www.ebi.ac.uk/interpro/) revealed that the amino acid sequence of AtBAG2 contains one UBL domain and one BD. Therefore, we investigated the roles of both of these domains in the molecular chaperone function of AtBAG2. First, we generated GST fusion constructs of three serial deletion derivatives of AtBAG2 (G-AtBAG2-Nt, G-AtBAG2-M, and G-AtBAG2-Ct) (Figure 4A). Among the three AtBAG2 deletion variants, G-AtBAG2-Nt contains the UBL domain, G-AtBAG2-M contains the BD, and G-AtBAG2-Ct contains no unique domain. The recombinant GST fusion proteins were then produced in *E. coli* and purified, and their hydrophobicities were compared with that of G-AtBAG2 using bis-ANS. Upon binding to bis-ANS, G-AtBAG2-M showed high fluorescence, as with G-AtBAG2, whereas G-AtBAG2-Nt and G-AtBAG2-Ct showed very low fluorescence (Figure 4B). We then compared the chaperone activities of G-AtBAG2-Nt, G-AtBAG2-M, and G-AtBAG2-Ct with that of G-AtBAG2 by measuring their ability to prevent the heat-induced denaturation of the substrate protein MDH. Light scattering increased rapidly when MDH (1.67 μM) was heated alone or with a 5-fold molar excess (8.35 μM) of G-AtBAG2-Nt and G-AtBAG2-Ct; however, when MDH (1.67 μM) was heated in the presence of a five-fold molar excess of G-AtBAG2 and G-AtBAG2-M, its aggregation was successively prevented (Figure 4C), confirming the molecular chaperone activity of G-AtBAG2-M and G-AtBAG2. Based on this information, we compared the effects of the overexpression of *G-AtBAG2*, *G-AtBAG2-Nt*, *G-AtBAG2-M*, and *G-AtBAG2-Ct* on the thermotolerance of yeast cells. The *G-AtBAG2-Nt*, *G-AtBAG2-M*, and *G-AtBAG2-Ct* genes were cloned under the control of the *GAL1* promoter to conditionally overexpress the recombinant proteins, and the constructs were named as *pYES2-G-AtBAG2-Nt*, *pYES2-G-AtBAG2-M*, and *pYES2-G-AtBAG2-Ct*, respectively. These three constructs as well as *pYES2-G-AtBAG2* and *pYES2-G* were separately transformed into the W303 cells. A spot assay was then performed using the heat-shocked *G*, *G-AtBAG2*, *G-AtBAG2-Nt*, *G-AtBAG2-M*, and *G-AtBAG2-Ct* transformants grown in media supplemented with 2% galactose (Gal) and sampled at the mid-exponential stage. The heat-shocked yeast cells overexpressing *G*, *G-AtBAG2-Nt*, and *G-AtBAG2-Ct* showed little difference in viability; by comparison, those overexpressing *G-AtBAG2* and *G-AtBAG2-M* showed significantly greater viability (Figure 4D). Taken together, these results suggest that the BD is critical for the molecular chaperone activity of AtBAG2, and overexpression of the BD-containing *AtBAG2* gene sequence significantly improves the viability of yeast cells under heat stress.

## 4. Discussion

The BAG family proteins, known to act as co-chaperones, share a well-conserved BD at their C-terminus, which facilitates their interaction with HSP70/HSC70 [1,5]. For example, the most representative BAG protein, human BAG1, functions as a nucleotide exchange factor (NEF) for the chaperone HSP70, and triggers the release of chaperone-bound proteins as a co-chaperone [26,27]. AtBAG1 also interacts with HSC70 via the BD [3], thus acting as a cofactor of HSC70 for the proteasomal degradation of unimported plastid proteins. However, it is difficult to find literature on BAG protein activity. According to the results of this study, AtBAG2 performs its own biochemical function without associating with other proteins. In particular, AtBAG2 was found to function as a molecular chaperone that prevents the heat- or chemical-induced aggregation of substrate proteins. AtBAG2 exhibited superior molecular chaperone activity with CS, which has frequently been used as a general substrate in molecular chaperone studies compared with Ypt1 (Figure 3B), which was previously identified as a molecular chaperone. In addition, AtBAG2 prevented the aggregation of thermally denatured MDH and chemically denatured insulin proteins, and its activity increased in a concentration-dependent manner (Figure 3C,D). Molecular chaperones exhibit high surface hydrophobicity [19,28], and AtBAG2 was no exception (Figure 3A). Based on these results, AtBAG2 was characterized as a novel molecular chaperone in this study.

To determine the region of AtBAG2 essential for its molecular chaperone activity, three deletion variants of the protein were generated, AtBAG2-Nt (UBL domain-containing N-terminal region), AtBAG2-Ct (domain-less C-terminal region), and AtBAG2-M (BD-containing middle region), and the activity of each variant protein was tested. Among the three derivative proteins, only AtBAG2-M exhibited molecular chaperone activity (Figure 4C), suggesting that the BD is responsible for the molecular chaperone function of AtBAG2. This suggests the possibility that the BD can act as a functionally active site in molecular chaperones, proposing new research directions and the applicability of BAG proteins in the future. In humans, the first and second helices of BAG1 BD are important for its binding to the serine/threonine kinase Raf-1 [29], while the second and third helices are known to mediate its binding to the ATPase domain of Hsc70/Hsp70 [30]. In the future, it will be interesting to find out which helices and amino acid residues in the BD of AtBAG2 are essential for the high hydrophobicity and molecular chaperone activity of the protein.

The maintenance of intracellular protein homeostasis is one of the most basic processes required for survival. Under stress conditions, intracellular proteins are often damaged and lose function, and molecular chaperones play a major role in maintaining life by preserving protein structure and function. For example, in humans, small heat shock proteins (sHsps), such as Hsp27, maintain cytoplasmic protein function and protect cells under stress conditions [31]. Ypt1p, a small GTPase in yeast, is generally responsible for intracellular trafficking; however, upon exposure to heat shock, Ypt1p undergoes structural changes which amplify its molecular chaperone function to protect cells [19]. In Arabidopsis, AtPP5 usually participates in cell signal transduction as a phosphatase; however, under thermal stress, it protects plants by acting as a molecular chaperone and preventing protein aggregation [32]. AtP3B, a ribosomal protein, exhibits high molecular chaperone activity, and its gene expression increases at high temperatures to protect Arabidopsis plants from heat-induced damage [28]. Similarly, AtBAG2 was found to exhibit excellent molecular chaperone activity in this study (Figure 3). Moreover, the *atbag2* mutant seedlings were sensitive to heat shock (Figure 1), whereas yeast cells overexpressing the *AtBAG2* gene showed enhanced survival under heat stress conditions (Figure 2), indicating that AtBAG2 enhances thermotolerance. Unfortunately, *AtBAG2* complementation lines in the *atbag2* mutant background could not be obtained, and therefore phenotypically characterized, in this study. Given that AtBAG2 enhances the tolerance to multiple abiotic stresses by regulating the expression of downstream genes involved in abscisic acid (ABA) response and stress-induced reactive oxygen species (ROS) production [13], revealing the relationship between its stress response mechanism and molecular chaperone activity is a research task that has yet to be accomplished. Our results, which identified and characterized AtBAG2 as a novel molecular chaperone, are expected to expand the research horizon for key factors such as HSP70 that regulate essential cellular functions by associating with BAG proteins.

## Data Availability

Data are contained within the article.

## Supplementary Materials

The following supporting information can be downloaded at: https://www.mdpi.com/article/10.3390/life13030687/s1, Figure S1: The original image for the Figure 1B (top); Figure S2: The original image for the Figure 1B (bottom); Figure S3: The original image for the Figure 1C (top); Figure S4: The original image for the Figure 1C (bottom).
