# Supplementary material for: Characterization of AtBAG2 as a Novel Molecular Chaperone
Source: Life (Basel). 2023 Mar 3;13(3):687. doi: 10.3390/life13030687 (PMC10052705; doi:10.3390/life13030687)
Supplement: Supplementary file 1 [file life-13-00687-s001.zip › life-2082097-supplementary.pdf]

Supplementary Materials:

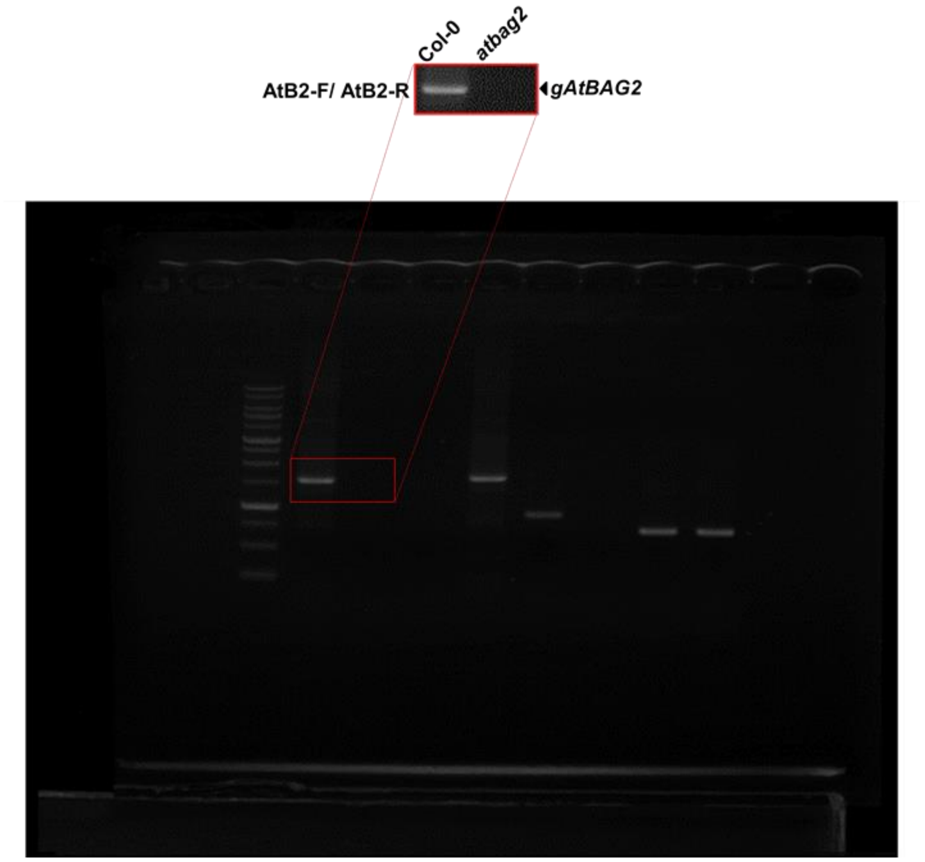

Figure S1. The original image for the Figure 1B (top). The area corresponding to Figure 1B(top) is indicated by a square with a red line on the original image.

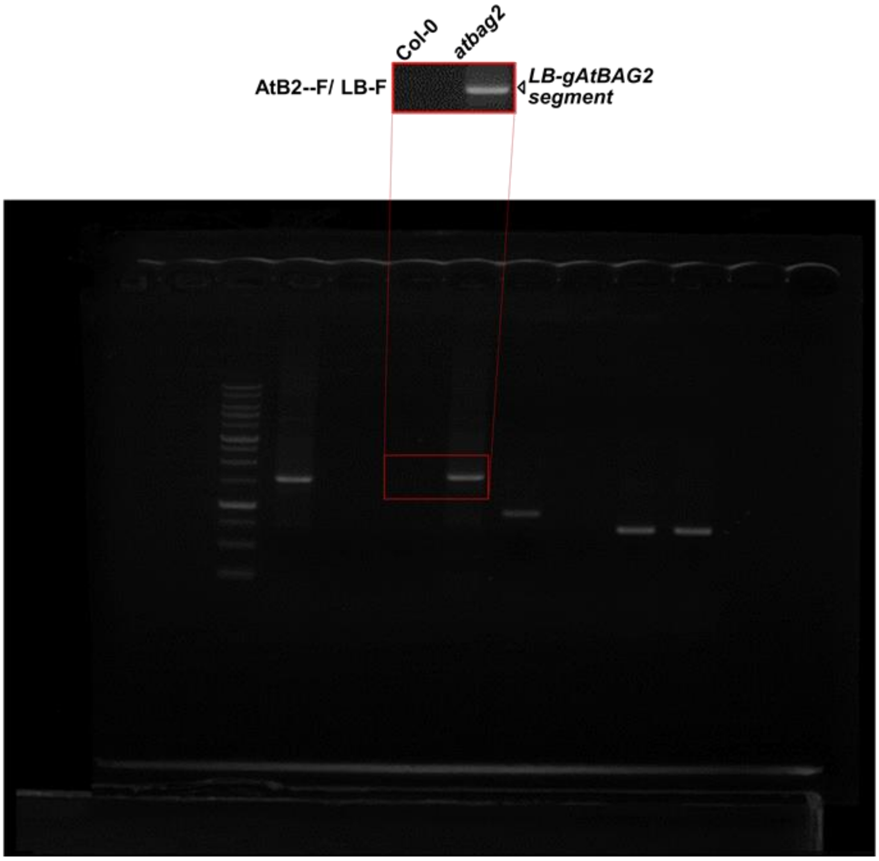

Figure S2. The original image for the Figure 1B (bottom). The area corresponding to Figure 1B (bottom) is indicated by a square with a red line on the original image.

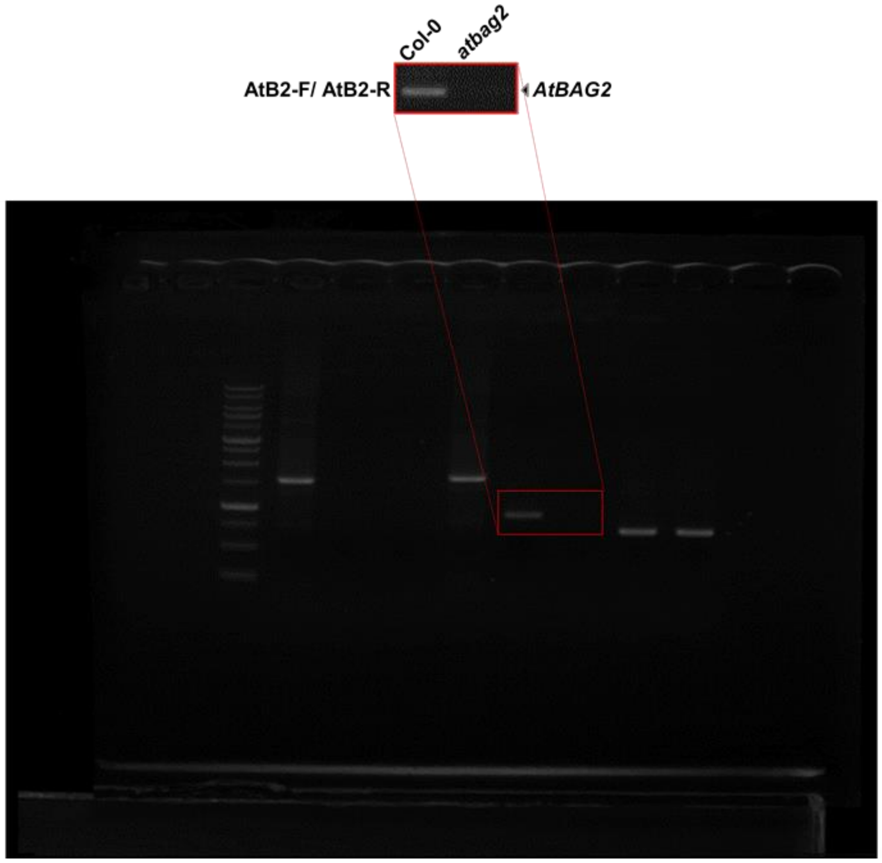

Figure S3. The original image for the Figure 1C (top). The area corresponding to Figure 1C (top) is indicated by a square with a red line on the original image.

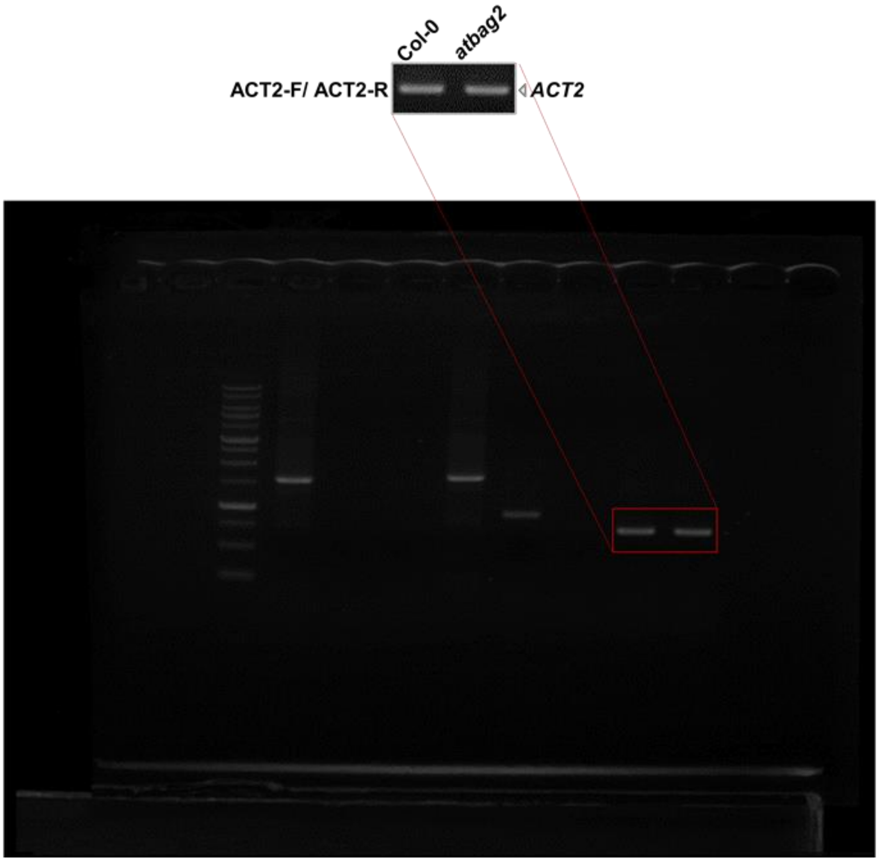

Figure S4. The original image for the Figure 1C (bottom). The area corresponding to Figure 1C (bottom) is indicated by a square with a red line on the original image.
